# Biogeographic models of gene flow in two waterfowl of the Australo-Papuan tropics

**DOI:** 10.1002/ece3.393

**Published:** 2012-10-09

**Authors:** David A Roshier, Robert Heinsohn, Gregory J Adcock, Peter Beerli, Leo Joseph

**Affiliations:** 1 Centre for Integrative Ecology, School of Life and Environmental Sciences, Deakin UniversityWaurn Ponds Campus, Geelong, VIC, 3217, Australia; 2 Fenner School of Environment and Society, Australian National UniversityCanberra, ACT, 0200, Australia; 3 School of Biology, Australian National UniversityCanberra, ACT, 0200, Australia; 4 Department of Scientific Computing, Florida State UniversityTallahassee, Florida; 5 Australian National Wildlife Collection, CSIRO Ecosystem SciencesGPO Box 1700, Canberra, ACT, 2601, Australia

**Keywords:** Anseriforms, genetic connectivity, Papua New Guinea, Australia

## Abstract

There are many large, easy-to-observe anseriform birds (ducks, geese, and swans) in northern Australia and New Guinea and they often gather in large numbers. Yet, the structure of their populations and their regional movements are poorly understood. Lack of understanding of population structure limits our capacity to understand source-sink dynamics relevant to their conservation or assess risks associated with avian-borne pathogens, in particular, avian influenza for which waterfowl are the main reservoir species. We set out to assess present-day genetic connectivity between populations of two widely distributed waterfowl in the Australo-Papuan tropics, magpie goose *Anseranas semipalmata* (Latham, 1798) and wandering whistling-duck *Dendrocygna arcuata* (Horsfield, 1824). Microsatellite data were obtained from 237 magpie geese and 64 wandering whistling-duck. Samples were collected across northern Australia, and at one site each in New Guinea and Timor Leste. In the wandering whistling-duck, genetic diversity was significantly apportioned by region and sampling location. For this species, the best model of population structure was New Guinea as the source population for all other populations. One remarkable result for this species was genetic separation of two flocks sampled contemporaneously on Cape York Peninsula only a few kilometers apart. In contrast, evidence for population structure was much weaker in the magpie goose, and Cape York as the source population provided the best fit to the observed structure. The fine scale genetic structure observed in wandering whistling-duck and magpie goose is consistent with earlier suggestions that the west-coast of Cape York Peninsula is a flyway for Australo-Papuan anseriforms between Australia and New Guinea across Torres Strait.

## Introduction

The evolution in isolation of Australo-Papua's distinctive avifauna is well known (Keast 1984; Ericson et al. 2002; Barker et al. 2004; Schodde 2006). Less widely appreciated is that within this region, there is substantial, ongoing isolation of much of the respective sub-avifaunas of Australia and New Guinea. Within the tropical parts of the region, many bird families are shared between the two land masses, however, at the species level, many birds are restricted range endemics confined to isolated habitats such as mountaintops in New Guinea (Mack and Dumbacher 2007) or rainforest remnants on Cape York Peninsula in northeastern Australia (Heinsohn and Legge 2003; Schodde 2006). Although <200 km apart, New Guinea and Australia share less than 15% of the 800+ species that occur in the region (Keast 1984). Recorded movements of birds across Torres Strait reflect either regular, seasonal movements of classically migratory species within Australo-Papua, irregular but frequent movements of individuals of vagile species, and infrequent movements of populations in response to events elsewhere, such as drought on mainland Australia (Draffan et al. 1983; Dingle 2004; Tracey et al. 2004).

Tropical northern Australia and the floodplains of southern New Guinea host a diverse anseriform avifauna (ducks, geese, and swans), of which eight species occur in both biomes (see Marchant and Higgins 1990; Halse et al. 1996; Bishop 2006). Most of these eight species breed throughout their range and are dispersive from their breeding sites in response to seasonal and/or irregular changes to wetland distribution (see Marchant and Higgins 1990). The few available banding records confirm that movements across Torres Strait have occurred in species such as the grey teal *Anas gracilis* (Frith 1982; Draffan et al. 1983), and some non-anseriform waterbirds (Geering et al. 1998), but the regularity and frequency of such movements remain speculative. As a result, the structure of waterfowl populations distributed across tropical areas of the Australo-Papuan region is unknown, as is the potential for waterfowl of Australian origin to mix on the floodplains of southern New Guinea with Palearctic waterfowl species that are possibly regular, but uncommon migrants or vagrants to the region (see Beehler et al. 1986; Marchant and Higgins 1990; Simpson and Day 2010). The latter is a concern in the context of the spread of avian-borne zoonotic diseases such as avian influenza (see McCallum et al. 2008; Klaassen et al. 2011).

**Figure 1:**
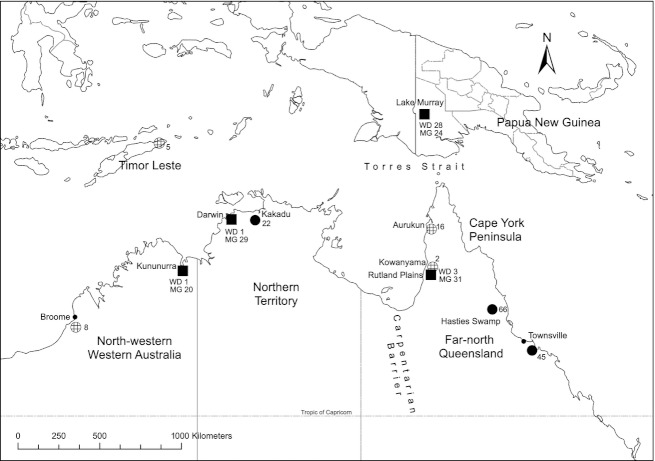
Regions and sites in Australia, Papua New Guinea, and Timor Leste mentioned in text and Table S1, and sample sizes per site for wandering whistling-duck *Dendrocygna arcuata* (*n* = 64) and magpie goose *Anseranas semipalmata* (*n* = 237). Solid circles are sites where only magpie goose were sampled and hatched circles are sites where only wandering whistling-duck were sampled.

Depending on a species' mobility, Torres Strait and its islands may act as either a bridge or barrier to birds that could occupy habitats on either side of the strait (Walker 1972). Such geographic features can result in recognizable patterns of genetic variation within and among populations such as that found in closed local populations, partially connected populations (meta-populations), or broad-scale homogeneity (panmixis) in populations for which such geographic features are not a barrier to gene flow (Avise 2000; Hellberg et al. 2002). In the same region, the Carpentarian Barrier (Fig. 1) is a tongue of sparsely vegetated tropical grassland and woodland extending south from the shores of the Gulf of Carpentaria. It separates the mesic forest and woodland environments of Cape York Peninsula to its east from those to its west in the Northern Territory and Western Australia (Macdonald 1969; Schodde and Mason 1999; Eldridge et al. 2011). Genetic studies have shown its differential role in shaping present-day genetic diversity in several bird species (Jennings and Edwards 2005; Kearns et al. 2010; Toon et al. 2010). Here, we seek to begin to clarify the movements and related population structure of anseriform birds across tropical regions of Australo-Papua. We studied in detail the magpie goose (*Anseranas semipalmata*) and wandering whistling-duck (*Dendrocygna arcuata*), both of which are known, at least anecdotally, to move between Australia and New Guinea (Ashford 1979; Draffan et al. 1983). Magpie geese are endemic to Australia and New Guinea and have no taxonomically recognized geographic variation (Marchant and Higgins 1990). They move seasonally between floodplains of northern Australia, where they spread widely during the wet season, and remnant wetlands in the dry season (Morton et al. 1990; Traill et al. 2010). Across northern Australia and New Guinea, there is one subspecies of wandering whistling-duck *D. arcuata australis* that differs only in size from two other currently recognized subspecies *D. arcuata arcuata* (Indonesia, Timor Leste, Philippines) and *D. arcuata pygmaea* (New Britain) (Marchant and Higgins 1990; Dickinson 2003). The movements of this species in Australo-Papua are poorly known, being either migratory or dispersive from dry season refuges (Marchant and Higgins 1990). The timing and duration of breeding in both species in northern Australia is dependent on the onset of the summer monsoon and the filling of suitable swamps, broadly this occurs from about December to April/May (Marchant and Higgins 1990).

No prior population-level genetic data exist for the two species examined in this study. We used rapidly evolving microsatellite loci to examine contemporary genetic processes and spatial patterns (see Burbrink 2010; Ciucchi and Gibbs 2010; Wang 2010) by comparing models of gene flow set in the biogeographic context of Australo-Papua.

## Materials and Methods

### Sample collection and DNA extraction

Two hundred and thirty-seven magpie goose and 64 wandering whistling-duck specimens were predominantly collected afresh by the authors and others (Appendix S1). Apart from some cryo-frozen tissue samples, most samples were blood taken from live birds captured in mist nets over 2 years from mid 2007. On Cape York Peninsula, we also used a CODA Netlauncher (CODA Enterprises, Inc., Mesa, Arizona) to target larger aggregations of anseriforms. Birds were sampled at multiple sites within the following regions (Fig. 1): Northwestern Australia (NWA), northernmost part (Top End) of the Northern Territory in the central part of northern Australia (NT), Cape York Peninsula in far northeastern Australia (CYP), Far North Queensland in lower northeastern Australia (FNQ), Papua New Guinea (PNG), and for wandering whistling-ducks in Timor Leste (TIM). The study populations represent two subspecies of the wandering whistling-duck *D. arcuata*. The TIM population is recognized as part of the nominotypical subspecies *D. a. arcuata*. All other studied populations represent *D. a. australis*. Differences in wing length diagnose these two subspecies (Mees 1975). No prior genetic evidence was available to distinguish TIM from the other populations. As subspecies, by definition, are not reproductively isolated, we included the TIM samples into our study of population model selection.

Blood was collected from the brachial vein, centrifuged to form serum and red blood cell fractions, and stored in ethanol, although some samples were received as whole blood in ethanol or on FTA© (Whatman, Maidstone, U.K.) cards. Cryo-frozen tissues were subsampled and stored in ethanol for transport to the laboratory. Samples were transported and stored at room temperature. DNA extraction methods largely followed Joseph et al. (2009). Extractions from tissue samples were done with DNeasy extraction kits (Qiagen, Valencia, CA) following the manufacturer's methods and from blood with the chelex method (adapted from Kline et al. 2002) with approximately 20-μL blood in ethanol or two, 4-mm holes punched from blood stored on FTA© cards. Samples on FTA© cards were first vortexed in 200 μL of Millipore purified water and left for 20 min before removing the solution. Chelex (150 μL, 5% w/v) was added to samples in 200-μL tubes and placed on a Corbett research PalmCycler for 20 min at 56°C before vortexing and incubating for 10 min at 99°C. DNA extracts were stored at −20°C.

### Screening of microsatellites

Forty-seven primer pairs previously shown to amplify polymorphic microsatellites in one or more anseriform birds were first tested in wandering whistling-ducks with amplification protocols based on Adcock and Mulder (2002) (primers chosen from Fields and Scribner 1997; Buchholz et al. 1998; Maak et al. 2003; Paulus and Tiedemann 2003; Guay and Mulder 2005; Huang et al. 2005). At least eight individuals were screened initially for each primer pair and those that produced unique and variable products were tested further. One primer in each pair had a 5′-M13 (TGTAAAACGACGGCCAGT) tail for use in the universal dye-labeling method described by Schuelke (2000). Seven loci were used for full screening: *MGgagt19, Smo6, Caud24, Caud4, Bcau10, Aph13, Blm3*. In magpie geese, the same protocol resulted in only *Caud24* and *Blm3* being suitable. Three further microsatellite loci, *MGgagt14, MGgagt19,* and *MG11*, were derived from a single library enriched for clones containing GA and GT repeats. These clones were constructed using DNA from one bird following Gardner et al. (1999) and modifications of Adcock and Mulder (2002). Of the 312 clones screened, primers were manufactured (Sigma-Aldrich, Castle Hill, New South Wales, Australia) for the 20 clones containing at least eight repeats and flanking sequence suitable for primer design. Microsatellites were scored using ABI GeneMapper software (Applied Biosystems, Foster City, California).

### Data analyses

Descriptive nucleotide diversity statistics and numbers of alleles were calculated using GenAlEx 6.0 (Peakall and Smouse 2006). Hardy–Weinberg Equilibrium (HWE) was tested using GenoDive 2.0 (Meirmans and Tienderen 2004). GenoDive uses an Analysis of Molecular Variance (AMOVA) procedure (Weir and Cockerham 1984) to calculate Φ_IS_ and thus test for HWE using a re-sampling procedure (9999 permutations were used). We present results calculated across all loci. Pairwise F_ST_ values were also calculated in GenoDive 2.0, which uses the Φ_ST_ value obtained from AMOVA (Excoffier et al. 1992) and this is analogous to the commonly used measures of Weir and Cockerham (1984). Again, 9999 permutations were done. Rarefaction analyses to account for sample size (Szpiech et al. 2008) were used to estimate the number of alleles expected in larger/older populations and private alleles (found only in a particular population possibly due to isolation from the others). We thus compared samples of different sizes with the sample with the smallest number of individuals. We used AMOVA to ask whether variation is significant among regions when compared with the within-region component. We also addressed whether variation between Australia and PNG is greater than that among regions within Australia. We used STRUCTURE (Pritchard et al. 2000; Pritchard and Wen 2004), a Bayesian clustering approach minimizing Hardy–Weinberg and linkage disequilibria, to test for geographic subdivision of regions and assignment of individuals to regions, to explore geographic structure in genotypic data for all individuals, and estimate *k*, the number of populations across all regions best supported by the data. We chose the number of populations where we observed the largest difference in log-likelihoods, ΔK (Evanno et al. 2005; see also Larsson et al. 2008).

Finally, we used a Bayes factor approach implemented in the program MIGRATE (Beerli 2006; Beerli and Palczewski 2010) to compare different biogeographic hypotheses for wandering whistling-duck and for magpie goose. The most general model allows for gene flow between all pairs of populations in both directions and has therefore the most parameters (25); a model that assumes that all sampling locations are part of a panmictic population needs only one parameter. At one site on CYP, we sampled wandering whistling-duck at two different localities 12.5 km and 1 week apart. We were particularly interested whether the flocks at these locations are independent of each other or represent a single, panmictic unit. These samples were treated separately in the analysis and hereafter termed Aurukun A and Aurukun B (Supplementary Information for sampling details).

For wandering whistling-duck, we evaluated the following five models: model I with PNG, Aurukun A, Aurukun B, NWA, TIM all connected permitting gene flow to all locations (20 mutation-scaled migration rates and five mutation-scaled population sizes are estimated); model II is the same as Model I, but the locations Aurukun A and Aurukun B are pooled (12 migration parameters, four population parameters); model III with PNG as the source population with direct migration routes to Aurukun A, Aurukun B, NWA, and TIM. The sink populations are not interconnected (five migration parameters, five population parameters); model IV is the same as model III, but the locations Aurukun A and Aurukun B are pooled (four migration parameters, four population parameters); In model V, all locations are part of a panmictic population (1 population parameter). For magpie goose, we evaluated the following seven models: model I with NWA, NT, CYP, FNQ, and PNG connected permitting gene flow among all locations. model II is the same as model I, but here, we pooled NWA and NT (NWA + NT) as biogeographic studies across northern Australia often find close relationships among populations in these two areas (Bowman et al. 2010); model III assumes that NT + NWA is the source and all other populations are sinks; model IV assumes that CYP is the source; model V assumes that FNQ is the source; and model VI assumes that PNG is the source; model VII finally assumes that all sampled magpie geese belong to a single panmictic population.

MIGRATE was run for each model using the microsatellite data; we used the Brownian mutation model (Beerli 2007). The MIGRATE run parameters were calibrated on the most complex Model I, so that the settings used for the comparison show convergence of the Markov chain Monte Carlo sampling method. We used the following settings for this comparison: the prior distributions were uniform for mutation-scaled population size parameters, that are four times the product of the effective population size and the mutation rate, and mutation-scaled migration rates M, that is, immigration rate scaled by the mutation rate, over the range of 0.0–50.0 and 0.0–80.0, respectively. Four independent chains using different acceptance ratios (temperature settings were 1.0; 1.5; 3.0; 1,000,000.0) were run concurrently. Each chain was a combination of 100 replicates, each of which discarded the first 10,000 samples as the burn-in. A total of 50 million states were visited and 50,000 states were recorded for the generation of posterior distribution histograms for each locus; for all loci, a total of 350 million states were visited and 350,000 samples were recorded. The different models were evaluated with marginal likelihoods. These were approximated with the Bézier-quadrature thermodynamic integration as described by Beerli and Palczewski (2010). The marginal likelihoods were then used to calculate Bayes factors and model probabilities using the formulas and model acceptance tables presented by Kass and Raftery (1995). For wandering whistling-duck, all samples from all locations were used, but for magpie goose, we ran each model five times and picked 20 randomly sampled individuals from each location. We then averaged the marginal likelihoods over these five runs. This procedure was chosen because the sampling of magpie geese was very uneven, making it difficult to get reliable runs from the full dataset.

## Results

All individuals sampled and the subsets of them screened for microsatellite data are in Table S1.

### Wandering whistling-duck

Specimens (*n* = 64) were screened from PNG (28), CYP (21), NT (1), NWA (9), and Timor Leste (5). The number of alleles per locus across all regions ranged from 2 (*Caud4*) to 19 (*Smo6*), whereas the mean number of alleles per region ranged from 1.71 ± 0.18 (NT) to 8.9 ± 2.26 (PNG). Rarefaction analyses (on all samples except NT) showed that although numbers of alleles are increasing with sample size in each region, differences are non-significant. Notably, though, the PNG value is continuing to rise. Locus-specific heterozygosity ranged from 0.094 (*Caud4*) to 0.844 (*Caud24*).

When all samples of wandering whistling-duck were pooled, HWE was not rejected (Φ_IS_ = 0.92, *P* = 0.062). The CYP samples of wandering whistling-duck, however, are not in HWE (Φ_IS_ = 0.086, *P* = 0.039) and that of Aurukun approaches significance (but note that the Aurukun A and B sample sizes are probably too small to test for HWE). Pairwise Φ_ST_ values are shown in Table 1. Almost all comparisons indicate significant apportioning of genetic diversity, whether compared by region or sampling site within regions and also when samples Aurukun A and B were separated. Notably, the value for the comparison of Aurukun A (CYP) to Lake Murray (PNG) was the lowest observed (0.006), and it was the only non-significant result where sample sizes were sufficient to detect differences. AMOVA further indicated substantial structuring of variation among regions when they were nested in the Australian and New Guinean landmasses (Φ_SC_ = 0.064 ± 0.024, *P* = 0.002). Not surprisingly, AMOVA with the Aurukun samples splits into A and B slightly reinforced this result with the *P* value changing from 0.002 to 0.000 (full AMOVA not shown).

**Table 1:** Summary of Φ_ST_ values (below diagonals) and associated *P* values (above, significant values in bold) in wandering whistling-duck *Dendrocygna arcuata* (Horsfield, 1824) by region (a); by sites, (b) and by sites with Aurukun samples separated (c). Italics indicate the only non-significant result where sample sizes were sufficient to detect differences (NT omitted due to low sample sizes)

	By region			
	
	CYP	NWA	PNG	Timor Leste
CYP	–	0.056	**0.006**	**0.001**
NWA	0.028	–	**0.009**	**0.005**
PNG	0.022	0.032	–	**0.006**
Timor Leste	0.106	0.134	0.055	

	By sites			
	
	PNG	Aurukun	NWA	Timor Leste
PNG	–	**0.005**	**0.007**	**0.003**
Aurukun	0.028	–	**0.015**	**0.001**
Broome	0.037	0.056	–	**0.006**
Timor Leste	0.055	0.119	0.141	–

	With Aurukun samples separated				
	
	PNG	Aurukun A	Aurukun B	NWA	Timor Leste
PNG	–	*0.256*	**0.000**	**0.008**	**0.002**
Aurukun A	*0.006*	–	**0.021**	0.053	**0.003**
Aurukun B	0.088	0.071	–	**0.007**	**0.007**
NWA	0.037	0.044	0.125	–	**0.006**
Timor Leste	0.056	0.098	0.200	0.141	–

CYP, Cape York Peninsula (Aurukun); NWA, Northwest Western Australia (Broome); PNG, Papua New Guinea (Lake Murray).

**Figure 2:**
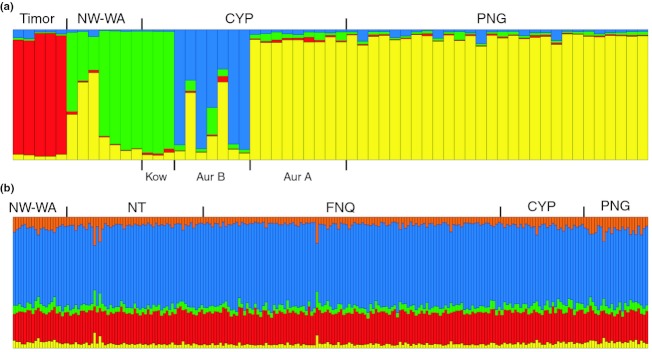
Output of STRUCTURE analysis in (A) wandering whistling-duck *Dendrocygna arcuata* showing regions from which samples were collected above the figure and localities on Cape York Peninsula below the figure, and (B) magpie goose *Anseranas semipalmata*. Regions are: Timor Leste (Timor), northwest Western Australia (NW-WA), Northern Territory (NT), Far North Queensland (FNQ), Cape York Peninsula (CYP), and Papua New Guinea (PNG). Localities on Cape York Peninsula are: Kowanyama (Kow), Aurukun A (Aur A), and Aurukun B (Aur B). See Figure 1 for all geographic locations.

STRUCTURE's estimates of the number of populations at *k* = 4 had the highest average log-likelihoods. An example of a run with *k* = 4 with log-likelihood of −1189 is shown in Figure 2. Delta log-likelihood values (ΔK) for *k* = 3 and *k* = 4 were similar at 27 and 23, respectively, but declined markedly at *k* ≥ 5. The additional population generated with *k* = 4 relative to *k* = 3 comprised only individuals from the Timor population. This is expected because the Timor population is a different subspecies. Our study affirms that this population is isolated from populations of *D. a. australis* in northern Australia and New Guinea. Thus, we conclude that *k =* 4 is optimal across all our samples.

Striking differentiation is evident among the samples from different sampling sites on CYP (Fig. 2A). STRUCTURE consistently partitioned the samples from these sites, such that Aurukun B was most distinct and Aurukun A shared diversity mostly with PNG. Subsequent analyses treated the two Aurukun samples as different flocks. Other CYP samples from south of Aurukun at Kowanyama are very different to both Aurukun A and B and grouped with NWA samples. The Kowanyama samples were not included in subsequent MIGRATE analyses because of small sample size.

The comparison of the five biogeographic hypotheses revealed that the Model III, which used PNG as a source population having migration routes into Aurukun A, Aurukun B, NWA, and TIM is favoured over all other tested models (Table 2). We can clearly rule out Model V, which assumes that birds sampled at all sites are members of the same panmictic population and Model I, which assumes that all sites exchange migrants according to an asymmetric n-island model. It is interesting that the Model III, which treats Aurukun A and Aurukun B as separate flocks, is ranked considerably higher than Model IV, despite the higher number of parameters. This suggests that Aurukun A and Aurukun B populations are derived from independent sources and dispersal events from PNG. A difference of 74 log units between Model III and Model IV is strong support for Model III using the Bayes factor and model acceptance tables (Table 2).

**Table 2:** Comparison of five biogeographic models for wandering whistling-duck *Dendrocygna arcuata* (Horsfield, 1824). Ln Bayes factor was calculated as the difference of the logarithms of the marginal likelihood of model III and all other models (Kass and Raftery 1995). For details see Methods. CYP is the combined location of Aurukun A and Aurukun B

Model	Description	Ln mL	Ln Bayes factor (Model III vs. Model I)	Model probability
I	PNG, Aurukun A, Aurukun B, NWA, TIM are all connected	−3113.78	1721.82	0.0000
II	PNG, CYP, NWA, TIM are all connected	−2761.69	1369.73	0.0000
III	PNG is the source Aurukun A, Aurukun B, NWA, and TIM are sinks	−1391.96	0.00	1.0000
IV	PNG is the source, CYP, NWA, and TIM are sinks	−1466.63	74.67	0.0000
V	PNG, Aurukun A, Aurukun B, NWA, and TIM are members of the same panmictic population	−2381.30	989.34	0.0000

CYP, Cape York Peninsula (Aurukun); NWA, Northwest Western Australia (Broome); PNG, Papua New Guinea (Lake Murray); TIM, Timor Leste; Ln mL, log marginal likelihood.

### Magpie goose

Specimens (*n* = 237) were obtained from PNG (24), CYP (31), NT (51), NWA (20), and FNQ (111). Between seven and nine alleles were observed at each locus in magpie goose and heterozygosity per locus ranged from 0.35 (*Blm3*) to 0.75 (*MGgagt14*). Mean numbers of alleles per region ranged from 4.60 ± 0.51(NWA) to 6.80 ± 0.37 (FNQ). Observed heterozygosity ranged from 0.58 ± 0.07 (NT) to 0.61 ± 0.08 (CYP), 0.63 ± 0.05 (PNG), and 0.63 ± 0.07 (NWA). Rarefaction analyses suggest that the PNG sample has more private alleles than others, but standard errors overlap substantially (data not shown). Hardy–Weinberg equilibrium across all loci was not rejected in any sample.

All pairwise comparisons of population differentiation by Φ_ST_ among regions and localities were non-significant (see Supplementary Material). The presence of private alleles in the PNG samples suggests caution here because the observed genetic variation is almost all within individuals, although a small, non-significant component was apportioned to regional differences. STRUCTURE suggests no subpopulation division: log-likelihoods of any estimate of the number of populations being greater than one were non-significant and no significant differences were detected among any samples at any level (Fig. 2B). The model comparison with MIGRATE reveals considerable patterns. model VII (panmixia) is clearly rejected and model IV is the best model tested. model IV uses the population on Cape York as a source and all other populations as sinks. The other models do represent the data better than the panmictic model, but do not explain the data well (Table 3).

**Table 3:** Comparison of seven biogeographic models for magpie goose *Anseranas semipalmata* (Latham 1798). Ln Bayes factor was calculated as the difference of the logarithms of the marginal likelihood of model IV and all other model (Kass and Raftery 1995). For details see Methods

Model	Description	Ln mL	Ln Bayes factor (Model IV vs. Model I)	Model probability
I	NWA, NT, CYP, FNQ, PNG	−2811	465	0.0000
II	NWA + NT, CYP, FNQ, PNG	−3461	1115	0.0000
III	NWA + NT is source and CYP, FNQ, and PNG are sinks	−2678	332	0.0000
IV	CYP is source and NWA + NT, FNQ, and PNG are sinks	−2346	0	1.0000
V	FNQ is source and NWA + NT, CYP, and PNG are sinks	−2898	552	0.0000
VI	PNG is source and NWA + NT, CYP, and FNQ are sinks	−2754	408	0.0000
VII	NWA, NT, CYP, FNQ, PNG belong to the same panmictic population	−7849	5503	0.0000

CYP, Cape York Peninsula (Aurukun); NWA, Northwest Western Australia (Broome); PNG, Papua New Guinea (Lake Murray); TIM, Timor Leste; Ln mL, log marginal likelihood.

## Discussion

This study set out to clarify population and genetic structure within and among the often-large populations of anseriform birds in the wetlands of northern Australia and New Guinea. Across northern Australia and New Guinea, populations of wandering whistling-duck and the magpie goose *appear* from standard texts (e.g., Marchant and Higgins 1990) to be disjunct. Our results suggest that both species show population structure, but that the connectivity among populations within each species is different and does not reflect a single biogeographic history shared by both species.

Each of the magpie goose populations we studied show similar numbers of alleles and similar expected heterozygosity. Allele frequency distributions and the model selection approach, however, revealed population structure. Caution is needed in interpreting this finding because only five loci could be examined and because of the presence of private alleles in the New Guinea samples. The latter result suggests that there may be more differentiation between Australia and New Guinea than we have been able to detect statistically. Nonetheless, Model IV best explained the observed variation in genetic diversity in magpie goose. This model suggests that the population on Cape York Peninsula is a source of variability and that all other populations receive migrants from it as a source. The n-island model with different migration rates, Model I, has rather low mutation-scaled migration rates, but the best model, Model IV, estimates rather high mutation-scaled migration rates among the populations. This may explain the inability of STRUCTURE to distinguish between a panmictic and a directional migration scenario (see also Larsson et al. 2008). This apparently high level of genetic connectivity may at first appear contrary to results from satellite tracking of 10 individuals of this species. That work showed the maximum linear distance that one bird moved in 38 weeks was only 114 km (Traill et al. 2010). However, such observations alone say nothing of where birds breed and thus where and when genes move. The models of observed allele frequency distributions presented here suggest that there is significant individual variation in movement responses, as has been observed in another Australian waterfowl – the grey teal (Roshier et al. 2008).

The observed genetic differentiation in wandering whistling-duck populations (or flocks) is remarkable in comparison to that observed in magpie goose populations that occupy the same habitats. The STRUCTURE result, in part, reflected the distinction between the two subspecies of WWD in our samples, *D. arcuata arcuata* from Timor Leste and all others, which belonged to *D. a. australis*. Of critical interest, however, was our finding that within the subspecies *D. a. australis,* the samples from two localities within the Aurukun site on Cape York Peninsula, and collected a week apart, were differentiated. Remarkably, birds captured at one of these localities, Aurukun A, were different to those from all other regions and sites apart from PNG, whereas those from Aurukun B were different from all others. The pattern of pairwise Φ_ST_ values in wandering whistling-duck coupled with the differences in the samples from Aurukun A and B could be explained in two ways: we may have sampled genetically divergent flocks that occur on Cape York Peninsula, or an immigrant flock from Papua New Guinea (see Beerli 2004). We evaluated population models in MIGRATE that pooled the Aurukun A and Aurukun B population into CYP and models that did not. The best model corroborates our STRUCTURE analysis. This suggests that Aurukun A and Aurukun B are not part of a single, panmictic population, but that all populations in Australia are connected to the population sampled in Papua New Guinea and that this population is a potential source of diversity across Australo-Papua. Wandering whistling-ducks certainly move as flocks and therefore may not be very well characterizable genetically by samples from any one geographic location. As our Aurukun A and B data show, this behavior could also mean that a given flock will not necessarily be similar genetically to other nearby flocks. Indeed, this result highlights a surprising dearth of genetic data from birds in which flocking behavior is typical, a characteristic evident in waterfowl and shorebirds perhaps more so than most landbirds. Specifically, there is a lack of data not just from multiple individuals captured within a single flock, but also from multiple flocks sampled multiple times at local and regional spatial scales (see Oomen et al. 2011 for an example). Notably, recent reviews (Anderson et al. 2010; Landguth et al. 2010) have highlighted specific aspects of this problem. They stressed the importance and intricacies of appropriate design for spatial and temporal sampling that is intended to assess gene flow. Our sampling, especially of wandering whistling-ducks, responded to some of these concerns. For example, spatial sampling should accommodate relationships among variables such as sampling grain and home range size. The hierarchical design to our sampling ranged over spatial scales from meters (within a flock) to about 10 km (between local flocks of wandering whistling-ducks) to 100 and 1000 km between sample sites across regions of northern Australia and Papua New Guinea. Similarly, landscape features exist at a broad range of spatial and temporal scales and our sampling recognized this. We sampled at multiple sites on either side of established biogeographic barriers that themselves have had dynamic histories. An example is our sampling at sites on either side of the Carpentarian Barrier, which is today represented by sea and sparsely wooded plains between Cape York Peninsula and the Northern Territory (Fig. 1; see Jennings and Edwards 2005; Kearns et al. 2010). If we are to improve our understanding of genetic diversity in these highly mobile species, there is a clear need for more systematically conducted surveys and careful analysis of how genetic diversity is apportioned within and between flocks distributed patchily on spatial scales as great as that as Cape York Peninsula (10,000 s km^2^). The genetic divergence between the two Aurukun A and B further affirms the value of sampling at smaller scales.

Wandering whistling-duck and magpie goose show considerable population structure and a model that assumes that the sampling locations are part of a large panmictic population can be excluded for both species. The best of the tested models, as estimated by Bayes factors, suggest a center of variability on Cape York Peninsula for the magpie goose and in Papua New Guinea for wandering whistling-ducks. One may assume that Cape York Peninsula represents a simple corridor. The presence there of differentiated populations in close geographic proximity to each other but differentiated genetically suggests that population structure is more complicated than can be explained by simple isolation-by-distance models, particularly in vagile species that flock.

Our findings also moderate any sense of isolation from avian-borne pathogens circulating in waterfowl populations in the archipelagos of Southeast Asia (Tracey et al. 2004; McCallum et al. 2008; Tracey 2010; Klaassen et al. 2011). The Australo-Papuan region is at the southern end of the East Asian-Australasian flyway. Of the 21 Palearctic waterfowls that annually migrate to eastern and southern Asia (Kear and Hulme 2005), only northern shoveler (*Anas clypeata*), northern pintail (*A. acuta*), and garganey (*A. querquedula*) are regular (although uncommon) migrants to the vast floodplains and coastal swamps of southern New Guinea (Beehler et al. 1986; Bishop 2006). In nearby northern Australia, Palearctic species mostly occur as vagrants along the northern coast of the continent during the summer monsoon (Marchant and Higgins 1990; Simpson and Day 2010), suggesting that there are long-standing strong ecological or physical barriers to the broader distribution of Palearctic waterfowl in the region. For the two waterfowl species examined in this study, the effects of distance over water, as a limit to gene flow, appear to occur at broad scales. The strongest differentiation we observed was that between *D. a. australis* of Australia and New Guinea and *D. a. arcuata* of Timor Leste, a distance of at least 550 km over water between adjacent populations. By contrast, the fine scale genetic structure observed in wandering whistling-duck and magpie goose is consistent with earlier suggestions that Cape York Peninsula, in particular the west-coast, is a flyway for Australo-Papuan anseriforms between Australia and New Guinea (Lavery 1970; Taplin 1991), thus potentially enabling Australian populations to mix with Palearctic species in southern New Guinea. This suggests that the short over water distance across Torres Strait is not a barrier to the movements of anseriforms in the region, although the context and frequency of passage likely vary markedly between species – as is evident in the two species studied here.

## Supporting Information

**Table S1.** Specimen codes of DNA extracts and corresponding locality and microsatellite data for all specimens studied here of wandering whistling-duck and magpie goose.

**Table S2.** Summary of Φ_ST_ values and associated *P* values in magpie goose *Anseranas semipalmata*.
