# Supplementary material for: Biogeographic models of gene flow in two waterfowl of the Australo-Papuan tropics
Source: Ecol Evol. 2012 Oct 9;2(11):2803–14. doi: 10.1002/ece3.393 (PMC3501632; doi:10.1002/ece3.393)
Supplement: Supplementary file 1 [file ece30002-2803-SD1.docx]

Table S1. Specimen codes of DNA extracts held at the Australian National Wildlife Collection (ANWC), CSIRO Ecosystem Sciences, Canberra, localities as shown in Figure 1 of the main paper and microsatellite data for wandering whistling-ducks and magpie geese studied in this paper. Wandering whistling-duck: Specimen codes, locality data and microsatellite data. Localities and regions are as shown in Figure 1 of main text. Regions: PNG – Papua New Guinea; CYP – Cape York Peninsula; NT- Northern Territory; NWA – North-west Western Australia; TIM – Timor Leste. Decimalized latitudes and longitudes: Lake Murray: -7.00, 141.5; Darwin area: -12.46, 130.84; Kowanyama: -15.33, 141.79; Aurukun A and B: -13.60, 141.82; Rutland Plains: -15.63, 141.82; Broome: -17.96, 122.24; Kununurra: -15.77, 128.74; Timor Leste. Samples with the prefix XX are taken from museum specimens held at the ANWC. All other samples collected afresh.

| Specimen Code | Locality | Region | Microsatellite loci and allelic lengths (base pairs) | | | | | | | | | | | | | |
| --- | --- | --- | --- | --- | --- | --- | --- | --- | --- | --- | --- | --- | --- | --- | --- | --- |
|  |  |  | *MGgagt19* | | *Smo6* | | *Caud24* | | *Caud4* | | *Bcau10* | | *Aph13* | | *Blm3* | |
| 07WW001 | Lake Murray | PNG | 120 | 126 | 245 | 253 | 178 | 186 | 219 | 219 | 118 | 124 | 185 | 186 | 180 | 188 |
| 07WW002 | Lake Murray | PNG | 120 | 126 | 241 | 245 | 184 | 184 | 219 | 219 | 118 | 122 | 186 | 187 | 182 | 188 |
| 07WW003 | Lake Murray | PNG | 120 | 120 | 243 | 247 | 179 | 186 | 219 | 219 | 122 | 122 | 185 | 187 | 180 | 193 |
| 07WW004 | Lake Murray | PNG | 120 | 126 | 243 | 256 | 187 | 187 | 219 | 219 | 122 | 122 | 187 | 187 | 180 | 183 |
| 07WW005 | Lake Murray | PNG | 120 | 120 | 240 | 255 | 187 | 188 | 219 | 219 | 122 | 122 | 188 | 189 | 180 | 182 |
| 07WW006 | Lake Murray | PNG | 120 | 120 | 246 | 253 | 187 | 190 | 219 | 219 | 118 | 122 | 186 | 186 | 181 | 187 |
| 07WW007 | Lake Murray | PNG | 120 | 126 | 245 | 253 | 182 | 186 | 219 | 219 | 118 | 124 | 182 | 186 | 183 | 190 |
| 07WW008 | Lake Murray | PNG | 120 | 126 | 239 | 245 | 186 | 187 | 219 | 219 | 118 | 128 | 185 | 188 | 178 | 181 |
| 07WW009 | Lake Murray | PNG | 120 | 120 | 241 | 242 | 185 | 186 | 212 | 219 | 118 | 122 | 182 | 185 | 180 | 182 |
| 07WW010 | Lake Murray | PNG | 120 | 120 | 253 | 253 | 185 | 186 | 212 | 219 | 118 | 118 | 186 | 187 | 178 | 182 |
| 07WW011 | Lake Murray | PNG | 120 | 120 | 241 | 242 | 186 | 187 | 219 | 219 | 118 | 118 | 182 | 186 | 182 | 183 |
| 07WW012 | Lake Murray | PNG | 120 | 126 | 241 | 260 | 187 | 192 | 219 | 219 | 118 | 122 | 185 | 186 | 180 | 183 |
| 07WW013 | Lake Murray | PNG | 120 | 126 | 245 | 245 | 184 | 187 | 219 | 219 | 122 | 122 | 186 | 186 | 181 | 192 |
| 07WW014 | Lake Murray | PNG | 120 | 120 | 244 | 253 | 186 | 187 | 219 | 219 | 118 | 118 | 182 | 182 | 180 | 180 |
| 07WW015 | Lake Murray | PNG | 120 | 120 | 238 | 243 | 186 | 188 | 219 | 219 | 118 | 122 | 187 | 187 | 179 | 181 |
| 07WW016 | Lake Murray | PNG | 120 | 126 | 241 | 249 | 185 | 185 | 219 | 219 | 122 | 122 | 182 | 185 | 181 | 191 |
| 07WW017 | Lake Murray | PNG | 120 | 126 | 240 | 248 | 180 | 193 | 219 | 219 | 118 | 122 | 185 | 185 | 181 | 186 |
| 07WW018 | Lake Murray | PNG | 120 | 126 | 241 | 253 | 184 | 189 | 219 | 219 | 118 | 122 | 186 | 187 | 184 | 187 |
| 07WW019 | Lake Murray | PNG | 120 | 126 | 238 | 241 | 187 | 187 | 219 | 219 | 118 | 122 | 182 | 182 | 180 | 181 |
| 07WW020 | Lake Murray | PNG | 120 | 126 | 240 | 253 | 185 | 189 | 219 | 219 | 122 | 122 | 185 | 185 | 187 | 190 |
| 07WW021 | Lake Murray | PNG | 126 | 126 | 248 | 260 | 177 | 186 | 219 | 219 | 118 | 120 | 187 | 187 | 181 | 181 |
| 07WW022 | Lake Murray | PNG | 126 | 126 | 239 | 260 | 186 | 189 | 219 | 219 | 118 | 122 | 186 | 188 | 183 | 191 |
| 07WW023 | Lake Murray | PNG | 120 | 126 | 240 | 241 | 187 | 190 | 219 | 219 | 118 | 122 | 185 | 187 | 179 | 183 |
| 07WW024 | Lake Murray | PNG | 120 | 121 | 240 | 245 | 179 | 185 | 219 | 219 | 118 | 122 | 182 | 185 | 178 | 183 |
| 07WW025 | Lake Murray | PNG | 120 | 120 | 243 | 253 | 187 | 192 | 219 | 219 | 118 | 122 | 186 | 187 | 180 | 180 |
| 07WW026 | Lake Murray | PNG | 120 | 120 | 240 | 242 | 186 | 187 | 219 | 219 | 118 | 122 | 186 | 187 | 181 | 181 |
| 07WW027 | Lake Murray | PNG | 120 | 120 | 240 | 240 | 185 | 188 | 212 | 219 | 118 | 118 | 186 | 186 | 181 | 181 |
| 07WW028 | Lake Murray | PNG | 120 | 120 | 239 | 245 | 184 | 187 | 219 | 219 | 118 | 122 | 185 | 189 | 187 | 190 |
| XXWW004 | Darwin area | NT | 120 | 120 | 239 | 244 | 188 | 190 | 219 | 219 | 122 | 124 | 186 | 187 | 181 | 188 |
| XXWW006 | Kowanyama | CYP | 120 | 126 | 240 | 241 | 186 | 187 | 219 | 219 | 122 | 126 | 189 | 189 | 179 | 187 |
| XXWW007 | Kowanyama | CYP | 120 | 126 | 240 | 241 | 187 | 188 | 219 | 219 | 118 | 122 | 186 | 189 | 179 | 182 |
| 08WW001 | Aurukun A | CYP | 120 | 126 | 239 | 244 | 187 | 189 | 219 | 219 | 122 | 122 | 0 | 0 | 180 | 180 |
| 08WW002 | Aurukun A | CYP | 120 | 120 | 245 | 260 | 187 | 189 | 212 | 219 | 118 | 122 | 185 | 185 | 181 | 193 |
| 08WW003 | Aurukun A | CYP | 126 | 126 | 241 | 255 | 186 | 188 | 219 | 219 | 118 | 122 | 186 | 186 | 181 | 181 |
| 08WW004 | Aurukun A | CYP | 120 | 126 | 241 | 260 | 186 | 189 | 219 | 219 | 122 | 122 | 186 | 188 | 182 | 182 |
| 08WW005 | Aurukun A | CYP | 120 | 126 | 241 | 255 | 186 | 187 | 219 | 219 | 118 | 122 | 185 | 185 | 179 | 183 |
| 08WW006 | Aurukun A | CYP | 120 | 120 | 241 | 246 | 185 | 193 | 219 | 219 | 118 | 122 | 185 | 188 | 181 | 188 |
| 08WW007 | Aurukun A | CYP | 120 | 126 | 240 | 245 | 186 | 186 | 219 | 219 | 118 | 122 | 185 | 186 | 181 | 181 |
| 08WW008 | Aurukun A | CYP | 120 | 126 | 245 | 255 | 177 | 188 | 219 | 219 | 118 | 122 | 185 | 185 | 180 | 187 |
| 08WW009 | Aurukun A | CYP | 126 | 126 | 236 | 240 | 185 | 186 | 219 | 219 | 118 | 120 | 186 | 186 | 183 | 183 |
| 08WW010 | Aurukun B | CYP | 120 | 120 | 241 | 241 | 185 | 185 | 219 | 219 | 122 | 122 | 185 | 185 | 180 | 183 |
| 08WW011 | Aurukun B | CYP | 120 | 120 | 239 | 240 | 185 | 188 | 212 | 219 | 122 | 122 | 185 | 185 | 183 | 183 |
| 08WW012 | Aurukun B | CYP | 120 | 126 | 241 | 245 | 186 | 189 | 219 | 219 | 118 | 122 | 187 | 187 | 180 | 183 |
| 08WW013 | Aurukun B | CYP | 120 | 126 | 241 | 241 | 177 | 188 | 219 | 219 | 118 | 122 | 185 | 186 | 180 | 183 |
| 08WW014 | Aurukun B | CYP | 120 | 120 | 240 | 241 | 185 | 188 | 212 | 219 | 122 | 122 | 185 | 185 | 180 | 183 |
| 08WW015 | Aurukun B | CYP | 120 | 126 | 241 | 241 | 188 | 191 | 219 | 219 | 122 | 122 | 182 | 186 | 178 | 181 |
| 08WW016 | Aurukun B | CYP | 120 | 120 | 240 | 241 | 185 | 185 | 219 | 219 | 122 | 122 | 185 | 186 | 183 | 183 |
| 09WW001 | Rutland Plains | CYP | 120 | 120 | 241 | 241 | 186 | 186 | 219 | 219 | 118 | 122 | 182 | 186 | 181 | 188 |
| 09WW002 | Rutland Plains | CYP | 120 | 120 | 239 | 240 | 177 | 186 | 219 | 219 | 122 | 122 | 186 | 186 | 184 | 188 |
| 09WW003 | Rutland Plains | CYP | 120 | 120 | 241 | 245 | 177 | 186 | 219 | 219 | 122 | 122 | 186 | 186 | 184 | 188 |
| 09WW004 | Broome | NWA | 120 | 126 | 241 | 241 | 177 | 186 | 219 | 219 | 122 | 122 | 186 | 186 | 184 | 187 |
| 09WW005 | Broome | NWA | 120 | 120 | 240 | 244 | 177 | 177 | 219 | 219 | 122 | 122 | 186 | 186 | 180 | 184 |
| 09WW006 | Broome | NWA | 120 | 120 | 239 | 244 | 177 | 179 | 219 | 219 | 118 | 122 | 186 | 186 | 180 | 184 |
| 09WW007 | Broome | NWA | 120 | 126 | 244 | 253 | 177 | 186 | 219 | 219 | 118 | 122 | 186 | 186 | 181 | 187 |
| 09WW008 | Broome | NWA | 120 | 120 | 240 | 241 | 187 | 188 | 219 | 219 | 122 | 124 | 185 | 187 | 186 | 187 |
| 09WW009 | Broome | NWA | 120 | 126 | 241 | 253 | 179 | 186 | 219 | 219 | 118 | 122 | 185 | 199 | 179 | 187 |
| 09WW010 | Broome | NWA | 120 | 120 | 244 | 253 | 186 | 187 | 219 | 219 | 118 | 122 | 185 | 186 | 180 | 180 |
| 09WW010 | Broome | NWA | 120 | 120 | 244 | 253 | 186 | 187 | 219 | 219 | 118 | 122 | 185 | 186 | 180 | 180 |
| XXWW003 | Kununurra | NWA | 120 | 120 | 240 | 240 | 185 | 186 | 219 | 219 | 118 | 122 | 185 | 186 | 180 | 180 |
| 09WW012 | Timor Leste | TIM | 120 | 120 | 248 | 261 | 186 | 188 | 219 | 219 | 118 | 122 | 187 | 191 | 181 | 182 |
| 09WW013 | Timor Leste | TIM | 120 | 120 | 248 | 261 | 186 | 187 | 219 | 219 | 118 | 126 | 187 | 193 | 181 | 182 |
| 09WW014 | Timor Leste | TIM | 120 | 120 | 248 | 261 | 186 | 187 | 219 | 219 | 118 | 126 | 187 | 193 | 181 | 182 |
| 09WW015 | Timor Leste | TIM | 120 | 120 | 241 | 241 | 185 | 187 | 219 | 219 | 118 | 122 | 184 | 184 | 182 | 182 |
| 09WW016 | Timor Leste | TIM | 120 | 120 | 237 | 237 | 186 | 186 | 219 | 219 | 118 | 122 | 185 | 185 | 180 | 180 |

Table S1 cont’d. Magpie Goose: Specimen codes, locality data and microsatellite data. Localities and regions are as shown in Figure 1 of main text. Regions: CYP – Cape York Peninsula; FNQ – Far North Queensland; NWA – North-west Western Australia; NT – Northern Territory; PNG – Papua New Guinea. Decimalized latitudes and longitudes: Big Red Lily Lagoon: -15.08, 144.14; Rutland Plains: -15.63, 141.82; Hasties Swamp: -17.30, 145.48; Townsville: -19.26, 146.82; Kununurra: -15.77, 128.74; Kakadu: -12.27, 132.67; Darwin area: -12.46, 130.84; Lake Murray: -7.00, 141.5.

| Specimen Code | Locality | Region | Microsatellite loci and allelic lengths (base pairs) | | | | | | | | | |
| --- | --- | --- | --- | --- | --- | --- | --- | --- | --- | --- | --- | --- |
|  |  |  | *Caud24* | | *MGgagt14* | | *MGgagt19* | | *Blm3* | | *MG11* | |
| 07MG001 | Big Red Lily Lagoon | CYP | 162 | 163 | 285 | 293 | 131 | 131 | 173 | 174 | 146 | 146 |
| 07MG002 | Big Red Lily Lagoon | CYP | 163 | 164 | 301 | 309 | 131 | 131 | 173 | 173 | 146 | 146 |
| 07MG003 | Big Red Lily Lagoon | CYP | 162 | 162 | 285 | 293 | 133 | 133 | 173 | 173 | 140 | 148 |
| 07MG004 | Big Red Lily Lagoon | CYP | 163 | 164 | 277 | 293 | 131 | 133 | 173 | 174 | 142 | 146 |
| 07MG005 | Big Red Lily Lagoon | CYP | 163 | 163 | 285 | 309 | 131 | 131 | 174 | 175 | 146 | 152 |
| 07MG006 | Big Red Lily Lagoon | CYP | 162 | 162 | 301 | 309 | 131 | 131 | 173 | 173 | 146 | 146 |
| 07MG007 | Big Red Lily Lagoon | CYP | 163 | 164 | 293 | 293 | 131 | 131 | 173 | 174 | 148 | 148 |
| 07MG008 | Big Red Lily Lagoon | CYP | 161 | 164 | 285 | 293 | 133 | 135 | 173 | 173 | 144 | 146 |
| 07MG009 | Big Red Lily Lagoon | CYP | 162 | 163 | 293 | 293 | 131 | 131 | 173 | 173 | 130 | 146 |
| 07MG010 | Big Red Lily Lagoon | CYP | 162 | 165 | 257 | 293 | 129 | 131 | 173 | 173 | 140 | 148 |
| 07MG012 | Big Red Lily Lagoon | CYP | 163 | 166 | 285 | 293 | 131 | 131 | 173 | 173 | 142 | 148 |
| 07MG013 | Big Red Lily Lagoon | CYP | 163 | 163 | 257 | 293 | 131 | 131 | 173 | 173 | 146 | 146 |
| 07MG014 | Big Red Lily Lagoon | CYP | 163 | 164 | 285 | 293 | 131 | 133 | 174 | 174 | 146 | 152 |
| 07MG015 | Big Red Lily Lagoon | CYP | 162 | 162 | 277 | 301 | 131 | 133 | 173 | 173 | 148 | 148 |
| 07MG016 | Big Red Lily Lagoon | CYP | 163 | 164 | 257 | 293 | 131 | 131 | 173 | 173 | 146 | 148 |
| 07MG017 | Big Red Lily Lagoon | CYP | 163 | 164 | 277 | 293 | 133 | 133 | 173 | 173 | 146 | 146 |
| 07MG018 | Big Red Lily Lagoon | CYP | 164 | 166 | 277 | 293 | 131 | 131 | 173 | 174 | 144 | 146 |
| 07MG019 | Big Red Lily Lagoon | CYP | 163 | 166 | 285 | 285 | 133 | 137 | 169 | 173 | 142 | 146 |
| 07MG020 | Big Red Lily Lagoon | CYP | 163 | 163 | 277 | 293 | 131 | 131 | 173 | 174 | 144 | 146 |
| 07MG021 | Big Red Lily Lagoon | CYP | 163 | 164 | 285 | 285 | 131 | 131 | 173 | 174 | 144 | 150 |
| 07MG022 | Big Red Lily Lagoon | CYP | 163 | 164 | 285 | 293 | 131 | 131 | 173 | 174 | 142 | 142 |
| 07MG023 | Big Red Lily Lagoon | CYP | 163 | 163 | 257 | 277 | 131 | 133 | 173 | 173 | 146 | 146 |
| 07MG024 | Big Red Lily Lagoon | CYP | 163 | 165 | 293 | 309 | 131 | 133 | 173 | 173 | 142 | 148 |
| 07MG025 | Big Red Lily Lagoon | CYP | 0 | 0 | 285 | 285 | 131 | 133 | 173 | 173 | 146 | 148 |
| 07MG026 | Big Red Lily Lagoon | CYP | 162 | 166 | 285 | 301 | 131 | 133 | 173 | 173 | 142 | 148 |
| 07MG027 | Big Red Lily Lagoon | CYP | 0 | 0 | 257 | 293 | 131 | 131 | 173 | 173 | 142 | 146 |
| 08MG001 | Big Red Lily Lagoon | CYP | 162 | 164 | 277 | 285 | 131 | 131 | 173 | 173 | 146 | 146 |
| 08MG002 | Big Red Lily Lagoon | CYP | 163 | 165 | 257 | 277 | 131 | 131 | 173 | 174 | 142 | 146 |
| 09MG009 | Rutland Plains | CYP | 163 | 164 | 257 | 285 | 131 | 133 | 169 | 173 | 146 | 146 |
| 09MG010 | Rutland Plains | CYP | 163 | 165 | 257 | 293 | 129 | 131 | 173 | 174 | 142 | 146 |
| 09MG011 | Rutland Plains | CYP | 164 | 164 | 285 | 293 | 131 | 131 | 173 | 173 | 146 | 148 |
| 07MG028 | Hasties Swamp | FNQ | 163 | 163 | 285 | 285 | 131 | 135 | 173 | 173 | 146 | 146 |
| 07MG029 | Hasties Swamp | FNQ | 163 | 166 | 277 | 285 | 131 | 133 | 173 | 174 | 142 | 146 |
| 07MG030 | Hasties Swamp | FNQ | 162 | 162 | 257 | 293 | 133 | 133 | 174 | 174 | 144 | 146 |
| 07MG031 | Hasties Swamp | FNQ | 162 | 162 | 257 | 285 | 131 | 133 | 173 | 173 | 142 | 148 |
| 07MG032 | Hasties Swamp | FNQ | 162 | 164 | 285 | 293 | 131 | 131 | 173 | 174 | 146 | 148 |
| 07MG033 | Hasties Swamp | FNQ | 163 | 163 | 277 | 293 | 131 | 131 | 173 | 173 | 146 | 148 |
| 07MG034 | Hasties Swamp | FNQ | 162 | 166 | 285 | 293 | 131 | 133 | 173 | 174 | 146 | 148 |
| 07MG035 | Hasties Swamp | FNQ | 163 | 163 | 285 | 285 | 131 | 133 | 173 | 173 | 146 | 148 |
| 07MG036 | Hasties Swamp | FNQ | 163 | 166 | 277 | 285 | 131 | 131 | 174 | 174 | 146 | 146 |
| 07MG037 | Hasties Swamp | FNQ | 163 | 164 | 285 | 293 | 131 | 131 | 173 | 174 | 140 | 146 |
| 07MG038 | Hasties Swamp | FNQ | 162 | 163 | 285 | 293 | 131 | 133 | 173 | 173 | 146 | 148 |
| 07MG039 | Hasties Swamp | FNQ | 163 | 164 | 285 | 293 | 131 | 135 | 173 | 174 | 142 | 146 |
| 07MG040 | Hasties Swamp | FNQ | 163 | 164 | 277 | 293 | 131 | 131 | 173 | 175 | 146 | 146 |
| 07MG041 | Hasties Swamp | FNQ | 163 | 164 | 285 | 293 | 131 | 131 | 173 | 173 | 142 | 146 |
| 07MG042 | Hasties Swamp | FNQ | 161 | 163 | 0 | 0 | 131 | 131 | 173 | 173 | 146 | 152 |
| 07MG043 | Hasties Swamp | FNQ | 163 | 163 | 293 | 309 | 131 | 135 | 173 | 174 | 142 | 146 |
| 07MG044 | Hasties Swamp | FNQ | 162 | 163 | 277 | 301 | 131 | 135 | 173 | 173 | 146 | 146 |
| 07MG045 | Hasties Swamp | FNQ | 163 | 166 | 285 | 285 | 129 | 135 | 174 | 174 | 146 | 146 |
| 07MG046 | Hasties Swamp | FNQ | 161 | 163 | 293 | 293 | 133 | 135 | 173 | 174 | 150 | 152 |
| 07MG047 | Hasties Swamp | FNQ | 165 | 166 | 285 | 293 | 131 | 133 | 173 | 173 | 140 | 146 |
| 07MG048 | Hasties Swamp | FNQ | 163 | 163 | 257 | 277 | 131 | 131 | 173 | 173 | 146 | 148 |
| 07MG049 | Hasties Swamp | FNQ | 0 | 0 | 285 | 293 | 131 | 133 | 173 | 173 | 146 | 146 |
| 07MG050 | Hasties Swamp | FNQ | 164 | 166 | 285 | 293 | 133 | 135 | 173 | 173 | 148 | 148 |
| 07MG051 | Hasties Swamp | FNQ | 163 | 163 | 277 | 293 | 131 | 131 | 173 | 174 | 148 | 150 |
| 07MG052 | Hasties Swamp | FNQ | 163 | 165 | 285 | 301 | 133 | 135 | 173 | 173 | 146 | 146 |
| 07MG054 | Hasties Swamp | FNQ | 163 | 166 | 285 | 293 | 131 | 135 | 173 | 173 | 146 | 146 |
| 07MG055 | Hasties Swamp | FNQ | 164 | 165 | 0 | 0 | 131 | 133 | 173 | 173 | 146 | 146 |
| 07MG056 | Hasties Swamp | FNQ | 161 | 162 | 0 | 0 | 131 | 131 | 173 | 174 | 142 | 146 |
| 07MG057 | Hasties Swamp | FNQ | 163 | 164 | 285 | 285 | 131 | 131 | 173 | 174 | 148 | 152 |
| 07MG058 | Hasties Swamp | FNQ | 163 | 164 | 301 | 309 | 131 | 131 | 173 | 174 | 146 | 146 |
| 07MG059 | Hasties Swamp | FNQ | 163 | 164 | 285 | 293 | 131 | 135 | 173 | 174 | 142 | 144 |
| 07MG060 | Hasties Swamp | FNQ | 162 | 164 | 277 | 285 | 131 | 133 | 173 | 174 | 142 | 146 |
| 07MG061 | Hasties Swamp | FNQ | 163 | 165 | 257 | 285 | 133 | 135 | 173 | 173 | 148 | 148 |
| 07MG062 | Hasties Swamp | FNQ | 163 | 163 | 285 | 293 | 131 | 131 | 173 | 173 | 146 | 146 |
| 07MG063 | Hasties Swamp | FNQ | 164 | 165 | 285 | 309 | 131 | 133 | 173 | 173 | 146 | 150 |
| 07MG064 | Hasties Swamp | FNQ | 163 | 166 | 285 | 293 | 131 | 133 | 173 | 174 | 144 | 146 |
| 07MG065 | Hasties Swamp | FNQ | 162 | 163 | 257 | 285 | 131 | 131 | 173 | 173 | 142 | 146 |
| 07MG066 | Hasties Swamp | FNQ | 163 | 166 | 265 | 285 | 129 | 135 | 173 | 174 | 146 | 146 |
| 07MG067 | Hasties Swamp | FNQ | 164 | 164 | 277 | 285 | 131 | 133 | 173 | 173 | 142 | 146 |
| 07MG068 | Hasties Swamp | FNQ | 163 | 163 | 0 | 0 | 131 | 131 | 173 | 173 | 146 | 146 |
| 07MG069 | Hasties Swamp | FNQ | 163 | 165 | 285 | 285 | 131 | 131 | 173 | 175 | 146 | 146 |
| 07MG070 | Hasties Swamp | FNQ | 163 | 164 | 0 | 0 | 131 | 133 | 173 | 173 | 146 | 150 |
| 07MG071 | Hasties Swamp | FNQ | 163 | 165 | 285 | 285 | 129 | 133 | 173 | 173 | 142 | 146 |
| 07MG072 | Hasties Swamp | FNQ | 163 | 163 | 277 | 285 | 131 | 131 | 173 | 173 | 146 | 152 |
| 07MG073 | Hasties Swamp | FNQ | 163 | 163 | 285 | 293 | 131 | 133 | 173 | 173 | 146 | 146 |
| 07MG074 | Hasties Swamp | FNQ | 163 | 163 | 277 | 285 | 131 | 133 | 173 | 173 | 130 | 148 |
| 07MG075 | Hasties Swamp | FNQ | 162 | 163 | 293 | 309 | 131 | 131 | 174 | 174 | 140 | 148 |
| 07MG076 | Hasties Swamp | FNQ | 163 | 166 | 285 | 293 | 131 | 133 | 173 | 173 | 146 | 152 |
| 07MG077 | Hasties Swamp | FNQ | 163 | 166 | 293 | 309 | 131 | 131 | 173 | 173 | 142 | 146 |
| 07MG078 | Hasties Swamp | FNQ | 164 | 164 | 277 | 285 | 131 | 133 | 173 | 173 | 142 | 150 |
| 07MG079 | Hasties Swamp | FNQ | 163 | 165 | 277 | 285 | 131 | 133 | 173 | 173 | 146 | 150 |
| 07MG080 | Hasties Swamp | FNQ | 163 | 166 | 277 | 285 | 131 | 131 | 173 | 173 | 146 | 146 |
| 07MG081 | Hasties Swamp | FNQ | 163 | 163 | 277 | 293 | 133 | 133 | 173 | 173 | 140 | 146 |
| 07MG082 | Hasties Swamp | FNQ | 162 | 163 | 285 | 293 | 131 | 133 | 174 | 174 | 146 | 152 |
| 07MG083 | Hasties Swamp | FNQ | 163 | 163 | 257 | 293 | 131 | 133 | 173 | 174 | 146 | 146 |
| 07MG084 | Hasties Swamp | FNQ | 162 | 164 | 285 | 285 | 133 | 133 | 173 | 173 | 144 | 146 |
| 07MG085 | Hasties Swamp | FNQ | 162 | 166 | 0 | 0 | 131 | 133 | 173 | 174 | 142 | 146 |
| 07MG086 | Hasties Swamp | FNQ | 163 | 166 | 293 | 293 | 131 | 133 | 173 | 173 | 146 | 152 |
| 07MG087 | Hasties Swamp | FNQ | 163 | 164 | 293 | 293 | 133 | 135 | 173 | 173 | 146 | 148 |
| 07MG088 | Hasties Swamp | FNQ | 163 | 163 | 285 | 309 | 131 | 131 | 173 | 173 | 146 | 146 |
| 07MG121 | Hasties Swamp | FNQ | 162 | 164 | 265 | 293 | 0 | 0 | 173 | 173 | 146 | 150 |
| 07MG122 | Hasties Swamp | FNQ | 163 | 163 | 285 | 293 | 131 | 133 | 172 | 173 | 142 | 142 |
| 07MG123 | Hasties Swamp | FNQ | 163 | 164 | 285 | 293 | 131 | 135 | 173 | 174 | 142 | 146 |
| 07MG124 | Hasties Swamp | FNQ | 165 | 166 | 285 | 301 | 131 | 135 | 173 | 174 | 146 | 146 |
| 07MG125 | Hasties Swamp | FNQ | 163 | 168 | 293 | 309 | 131 | 131 | 173 | 173 | 142 | 146 |
| 07MG126 | Hasties Swamp | FNQ | 162 | 164 | 293 | 301 | 131 | 133 | 173 | 173 | 146 | 148 |
| 07MG223 | Townsville | FNQ | 0 | 0 | 277 | 277 | 131 | 131 | 173 | 173 | 148 | 148 |
| 07MG224 | Townsville | FNQ | 162 | 164 | 257 | 293 | 131 | 131 | 173 | 174 | 148 | 148 |
| 07MG225 | Townsville | FNQ | 0 | 0 | 0 | 0 | 121 | 127 | 172 | 173 | 142 | 148 |
| 07MG226 | Townsville | FNQ | 162 | 164 | 285 | 285 | 131 | 131 | 173 | 173 | 146 | 146 |
| 07MG227 | Townsville | FNQ | 164 | 164 | 285 | 293 | 131 | 131 | 173 | 173 | 0 | 0 |
| 07MG228 | Townsville | FNQ | 163 | 163 | 257 | 257 | 131 | 131 | 173 | 174 | 140 | 146 |
| 07MG229 | Townsville | FNQ | 164 | 164 | 285 | 285 | 131 | 131 | 173 | 173 | 146 | 152 |
| 07MG230 | Townsville | FNQ | 162 | 163 | 285 | 293 | 131 | 133 | 173 | 173 | 148 | 152 |
| 07MG231 | Townsville | FNQ | 163 | 164 | 257 | 293 | 131 | 135 | 173 | 174 | 146 | 146 |
| 07MG232 | Townsville | FNQ | 163 | 164 | 285 | 293 | 131 | 133 | 173 | 173 | 130 | 142 |
| 07MG233 | Townsville | FNQ | 162 | 163 | 277 | 293 | 131 | 135 | 174 | 174 | 142 | 148 |
| 07MG234 | Townsville | FNQ | 163 | 164 | 257 | 293 | 131 | 135 | 173 | 173 | 146 | 146 |
| 07MG235 | Townsville | FNQ | 162 | 163 | 285 | 285 | 131 | 133 | 173 | 175 | 142 | 146 |
| 07MG236 | Townsville | FNQ | 162 | 162 | 285 | 293 | 131 | 133 | 173 | 173 | 148 | 148 |
| 07MG237 | Townsville | FNQ | 162 | 164 | 285 | 293 | 131 | 133 | 173 | 173 | 146 | 146 |
| 07MG238 | Townsville | FNQ | 162 | 166 | 285 | 293 | 131 | 135 | 173 | 173 | 148 | 148 |
| 07MG239 | Townsville | FNQ | 163 | 163 | 285 | 293 | 131 | 131 | 174 | 174 | 146 | 148 |
| 07MG240 | Townsville | FNQ | 163 | 166 | 285 | 285 | 131 | 133 | 173 | 174 | 142 | 146 |
| 08MG003 | Townsville | FNQ | 162 | 163 | 293 | 293 | 131 | 131 | 173 | 173 | 146 | 148 |
| 08MG004 | Townsville | FNQ | 164 | 164 | 277 | 285 | 133 | 133 | 173 | 173 | 146 | 148 |
| 08MG005 | Townsville | FNQ | 163 | 164 | 285 | 293 | 131 | 133 | 173 | 173 | 146 | 150 |
| 08MG006 | Townsville | FNQ | 162 | 163 | 285 | 301 | 131 | 133 | 173 | 174 | 130 | 146 |
| 08MG007 | Townsville | FNQ | 163 | 164 | 257 | 285 | 131 | 131 | 173 | 173 | 146 | 146 |
| 08MG008 | Townsville | FNQ | 164 | 164 | 285 | 293 | 133 | 133 | 173 | 174 | 146 | 148 |
| 08MG009 | Townsville | FNQ | 163 | 164 | 277 | 285 | 131 | 131 | 173 | 173 | 148 | 148 |
| 08MG010 | Townsville | FNQ | 163 | 163 | 257 | 285 | 131 | 131 | 173 | 173 | 146 | 146 |
| 08MG011 | Townsville | FNQ | 163 | 164 | 277 | 293 | 131 | 133 | 169 | 174 | 140 | 146 |
| 08MG012 | Townsville | FNQ | 0 | 0 | 293 | 293 | 131 | 131 | 169 | 173 | 142 | 146 |
| 08MG013 | Townsville | FNQ | 0 | 0 | 257 | 293 | 129 | 131 | 169 | 173 | 146 | 146 |
| 08MG014 | Townsville | FNQ | 163 | 164 | 277 | 285 | 131 | 133 | 173 | 173 | 146 | 150 |
| 08MG015 | Townsville | FNQ | 163 | 163 | 257 | 285 | 131 | 131 | 173 | 174 | 146 | 146 |
| 08MG016 | Townsville | FNQ | 162 | 162 | 257 | 309 | 131 | 135 | 169 | 174 | 146 | 148 |
| 08MG017 | Townsville | FNQ | 164 | 164 | 285 | 293 | 131 | 133 | 169 | 173 | 146 | 146 |
| 08MG018 | Townsville | FNQ | 163 | 164 | 257 | 285 | 133 | 133 | 173 | 174 | 142 | 146 |
| 08MG019 | Townsville | FNQ | 162 | 164 | 277 | 285 | 133 | 133 | 171 | 173 | 142 | 142 |
| 08MG020 | Townsville | FNQ | 163 | 163 | 285 | 293 | 131 | 133 | 173 | 173 | 152 | 152 |
| 08MG021 | Townsville | FNQ | 162 | 163 | 257 | 285 | 131 | 131 | 173 | 174 | 144 | 152 |
| 08MG022 | Townsville | FNQ | 163 | 163 | 257 | 293 | 131 | 131 | 173 | 175 | 146 | 146 |
| 08MG023 | Townsville | FNQ | 162 | 166 | 277 | 285 | 131 | 133 | 173 | 173 | 144 | 152 |
| 08MG024 | Townsville | FNQ | 162 | 163 | 277 | 285 | 131 | 131 | 173 | 173 | 146 | 152 |
| 08MG025 | Townsville | FNQ | 163 | 163 | 285 | 285 | 131 | 133 | 173 | 174 | 146 | 152 |
| 08MG026 | Townsville | FNQ | 162 | 164 | 277 | 293 | 131 | 133 | 169 | 173 | 140 | 146 |
| 09MG006 | Townsville | FNQ | 163 | 164 | 257 | 285 | 131 | 135 | 173 | 173 | 140 | 146 |
| 09MG007 | Townsville | FNQ | 163 | 163 | 285 | 285 | 131 | 133 | 173 | 174 | 142 | 146 |
| 09MG008 | Townsville | FNQ | 166 | 166 | 293 | 293 | 131 | 133 | 173 | 173 | 146 | 148 |
| 07MG127 | Kununurra | NWA | 163 | 164 | 293 | 293 | 129 | 131 | 173 | 173 | 146 | 146 |
| 07MG128 | Kununurra | NWA | 164 | 164 | 285 | 285 | 131 | 131 | 173 | 173 | 146 | 148 |
| 07MG129 | Kununurra | NWA | 163 | 163 | 293 | 293 | 131 | 133 | 173 | 173 | 146 | 148 |
| 07MG130 | Kununurra | NWA | 162 | 164 | 293 | 293 | 131 | 135 | 173 | 173 | 142 | 148 |
| 07MG131 | Kununurra | NWA | 162 | 163 | 257 | 309 | 131 | 135 | 173 | 174 | 144 | 146 |
| 07MG132 | Kununurra | NWA | 162 | 163 | 257 | 285 | 131 | 133 | 0 | 0 | 142 | 144 |
| 07MG133 | Kununurra | NWA | 163 | 164 | 293 | 293 | 131 | 133 | 173 | 174 | 144 | 146 |
| 07MG134 | Kununurra | NWA | 163 | 164 | 277 | 293 | 131 | 133 | 178 | 181 | 144 | 152 |
| 07MG135 | Kununurra | NWA | 164 | 166 | 285 | 309 | 131 | 131 | 173 | 173 | 148 | 148 |
| 07MG136 | Kununurra | NWA | 162 | 164 | 285 | 301 | 129 | 131 | 173 | 173 | 146 | 146 |
| 07MG137 | Kununurra | NWA | 162 | 163 | 285 | 293 | 129 | 131 | 173 | 173 | 144 | 144 |
| 07MG138 | Kununurra | NWA | 162 | 166 | 293 | 309 | 131 | 133 | 0 | 0 | 142 | 148 |
| 07MG139 | Kununurra | NWA | 163 | 166 | 277 | 293 | 131 | 133 | 173 | 173 | 142 | 146 |
| 07MG140 | Kununurra | NWA | 163 | 165 | 285 | 309 | 131 | 131 | 173 | 173 | 146 | 146 |
| 07MG141 | Kununurra | NWA | 162 | 162 | 285 | 285 | 131 | 131 | 173 | 173 | 144 | 146 |
| 07MG142 | Kununurra | NWA | 163 | 164 | 285 | 293 | 131 | 131 | 173 | 173 | 146 | 146 |
| 07MG143 | Kununurra | NWA | 163 | 163 | 285 | 285 | 133 | 135 | 173 | 173 | 146 | 146 |
| 07MG144 | Kununurra | NWA | 163 | 166 | 277 | 309 | 131 | 131 | 173 | 173 | 146 | 148 |
| 07MG145 | Kununurra | NWA | 163 | 166 | 285 | 293 | 129 | 135 | 174 | 174 | 142 | 146 |
| 07MG146 | Kununurra | NWA | 163 | 166 | 277 | 293 | 133 | 133 | 173 | 173 | 142 | 144 |
| 07MG147 | Kakadu | NT | 163 | 163 | 285 | 293 | 131 | 131 | 173 | 173 | 146 | 146 |
| 07MG148 | Kakadu | NT | 163 | 164 | 285 | 293 | 131 | 135 | 173 | 175 | 146 | 146 |
| 07MG149 | Kakadu | NT | 164 | 166 | 285 | 293 | 131 | 131 | 173 | 173 | 142 | 146 |
| 07MG150 | Kakadu | NT | 163 | 164 | 277 | 301 | 133 | 135 | 173 | 173 | 140 | 146 |
| 07MG151 | Kakadu | NT | 163 | 164 | 285 | 293 | 131 | 131 | 173 | 173 | 146 | 146 |
| 07MG152 | Kakadu | NT | 163 | 164 | 257 | 293 | 133 | 133 | 173 | 173 | 146 | 152 |
| 07MG153 | Kakadu | NT | 163 | 163 | 293 | 309 | 131 | 133 | 173 | 173 | 146 | 146 |
| 07MG154 | Kakadu | NT | 163 | 163 | 293 | 293 | 131 | 131 | 173 | 174 | 142 | 144 |
| 07MG155 | Kakadu | NT | 163 | 163 | 257 | 257 | 131 | 133 | 173 | 174 | 146 | 146 |
| 07MG156 | Kakadu | NT | 163 | 163 | 257 | 285 | 131 | 131 | 173 | 173 | 142 | 146 |
| 07MG157 | Kakadu | NT | 163 | 164 | 277 | 285 | 131 | 135 | 173 | 174 | 146 | 148 |
| 07MG158 | Kakadu | NT | 162 | 164 | 293 | 309 | 129 | 133 | 173 | 173 | 146 | 148 |
| 07MG159 | Kakadu | NT | 164 | 166 | 293 | 293 | 131 | 135 | 173 | 174 | 146 | 148 |
| 07MG160 | Kakadu | NT | 163 | 163 | 285 | 293 | 131 | 131 | 0 | 0 | 146 | 148 |
| 07MG161 | Kakadu | NT | 163 | 163 | 285 | 293 | 131 | 133 | 173 | 173 | 148 | 150 |
| 07MG162 | Kakadu | NT | 163 | 164 | 285 | 293 | 131 | 133 | 173 | 174 | 142 | 148 |
| 07MG163 | Kakadu | NT | 163 | 163 | 293 | 293 | 129 | 133 | 173 | 174 | 146 | 146 |
| 07MG164 | Kakadu | NT | 163 | 166 | 285 | 285 | 131 | 131 | 173 | 173 | 146 | 146 |
| 07MG165 | Kakadu | NT | 163 | 163 | 285 | 293 | 131 | 135 | 173 | 173 | 144 | 148 |
| 07MG166 | Kakadu | NT | 162 | 164 | 285 | 293 | 131 | 131 | 173 | 173 | 142 | 148 |
| 07MG167 | Kakadu | NT | 163 | 163 | 285 | 285 | 131 | 131 | 173 | 173 | 146 | 146 |
| 07MG168 | Kakadu | NT | 0 | 0 | 277 | 285 | 131 | 133 | 173 | 173 | 146 | 150 |
| 07MG169 | Darwin area | NT | 163 | 164 | 293 | 293 | 131 | 131 | 173 | 173 | 146 | 152 |
| 07MG170 | Darwin area | NT | 164 | 164 | 285 | 285 | 129 | 131 | 173 | 173 | 142 | 146 |
| 07MG171 | Darwin area | NT | 163 | 166 | 277 | 285 | 131 | 131 | 173 | 173 | 142 | 146 |
| 07MG172 | Darwin area | NT | 163 | 163 | 257 | 285 | 131 | 131 | 173 | 173 | 146 | 148 |
| 07MG173 | Darwin area | NT | 163 | 164 | 277 | 301 | 131 | 133 | 173 | 175 | 142 | 146 |
| 07MG174 | Darwin area | NT | 163 | 165 | 285 | 293 | 131 | 133 | 173 | 173 | 146 | 148 |
| 07MG175 | Darwin area | NT | 163 | 166 | 277 | 285 | 131 | 135 | 173 | 173 | 142 | 146 |
| 07MG176 | Darwin area | NT | 163 | 164 | 257 | 285 | 133 | 137 | 173 | 174 | 146 | 148 |
| 07MG177 | Darwin area | NT | 162 | 163 | 257 | 257 | 131 | 133 | 173 | 174 | 146 | 146 |
| 07MG178 | Darwin area | NT | 163 | 163 | 277 | 293 | 133 | 135 | 173 | 173 | 146 | 146 |
| 07MG179 | Darwin area | NT | 163 | 163 | 277 | 285 | 0 | 0 | 173 | 174 | 146 | 148 |
| 07MG180 | Darwin area | NT | 163 | 163 | 277 | 293 | 131 | 133 | 173 | 173 | 142 | 142 |
| 07MG181 | Darwin area | NT | 162 | 163 | 293 | 309 | 133 | 133 | 173 | 173 | 142 | 146 |
| 07MG182 | Darwin area | NT | 163 | 164 | 285 | 285 | 131 | 135 | 173 | 174 | 142 | 150 |
| 07MG183 | Darwin area | NT | 163 | 163 | 285 | 293 | 131 | 131 | 173 | 173 | 146 | 148 |
| 07MG184 | Darwin area | NT | 163 | 166 | 257 | 293 | 133 | 135 | 173 | 175 | 144 | 148 |
| 07MG185 | Darwin area | NT | 163 | 163 | 0 | 0 | 121 | 131 | 173 | 174 | 144 | 148 |
| 07MG186 | Darwin area | NT | 163 | 164 | 293 | 301 | 131 | 131 | 173 | 173 | 140 | 146 |
| 07MG187 | Darwin area | NT | 163 | 163 | 0 | 0 | 121 | 121 | 173 | 173 | 142 | 144 |
| 07MG188 | Darwin area | NT | 163 | 163 | 285 | 293 | 131 | 133 | 173 | 173 | 146 | 146 |
| 07MG189 | Darwin area | NT | 163 | 165 | 285 | 293 | 131 | 131 | 173 | 173 | 146 | 146 |
| 07MG190 | Darwin area | NT | 163 | 166 | 0 | 0 | 133 | 133 | 173 | 173 | 146 | 146 |
| 07MG191 | Darwin area | NT | 163 | 164 | 269 | 285 | 131 | 131 | 173 | 173 | 146 | 146 |
| 07MG192 | Darwin area | NT | 163 | 165 | 285 | 285 | 131 | 131 | 173 | 173 | 146 | 152 |
| 07MG193 | Darwin area | NT | 163 | 164 | 277 | 277 | 0 | 0 | 173 | 173 | 0 | 0 |
| 07MG194 | Darwin area | NT | 163 | 163 | 277 | 293 | 133 | 133 | 173 | 173 | 0 | 0 |
| 07MG195 | Darwin area | NT | 163 | 166 | 277 | 285 | 131 | 133 | 173 | 173 | 0 | 0 |
| 07MG196 | Darwin area | NT | 163 | 164 | 277 | 285 | 0 | 0 | 173 | 173 | 142 | 148 |
| 07MG197 | Darwin area | NT | 162 | 164 | 293 | 301 | 131 | 131 | 173 | 174 | 146 | 148 |
| 07MG198 | Lake Murray | PNG | 163 | 166 | 277 | 293 | 131 | 133 | 173 | 173 | 142 | 146 |
| 07MG199 | Lake Murray | PNG | 163 | 166 | 285 | 285 | 131 | 133 | 173 | 173 | 144 | 146 |
| 07MG200 | Lake Murray | PNG | 163 | 163 | 257 | 257 | 129 | 131 | 173 | 174 | 146 | 146 |
| 07MG201 | Lake Murray | PNG | 163 | 163 | 293 | 293 | 131 | 131 | 173 | 174 | 142 | 152 |
| 07MG202 | Lake Murray | PNG | 164 | 165 | 285 | 293 | 131 | 131 | 173 | 174 | 142 | 146 |
| 07MG203 | Lake Murray | PNG | 164 | 164 | 277 | 293 | 131 | 131 | 173 | 173 | 146 | 146 |
| 07MG204 | Lake Murray | PNG | 163 | 163 | 285 | 293 | 131 | 131 | 173 | 173 | 142 | 146 |
| 07MG205 | Lake Murray | PNG | 163 | 163 | 277 | 277 | 131 | 132 | 173 | 173 | 144 | 146 |
| 07MG206 | Lake Murray | PNG | 164 | 165 | 285 | 285 | 131 | 131 | 173 | 173 | 146 | 148 |
| 07MG207 | Lake Murray | PNG | 163 | 164 | 277 | 285 | 131 | 135 | 173 | 174 | 142 | 146 |
| 07MG208 | Lake Murray | PNG | 163 | 166 | 285 | 293 | 131 | 133 | 173 | 173 | 142 | 146 |
| 07MG209 | Lake Murray | PNG | 164 | 164 | 285 | 285 | 131 | 131 | 173 | 173 | 148 | 150 |
| 07MG210 | Lake Murray | PNG | 164 | 166 | 285 | 285 | 131 | 133 | 173 | 173 | 144 | 146 |
| 07MG211 | Lake Murray | PNG | 162 | 162 | 285 | 285 | 131 | 135 | 173 | 173 | 146 | 146 |
| 07MG212 | Lake Murray | PNG | 164 | 164 | 277 | 293 | 131 | 133 | 173 | 173 | 142 | 146 |
| 07MG213 | Lake Murray | PNG | 162 | 163 | 293 | 293 | 131 | 133 | 173 | 173 | 144 | 146 |
| 07MG214 | Lake Murray | PNG | 162 | 166 | 285 | 285 | 131 | 133 | 173 | 173 | 146 | 146 |
| 07MG215 | Lake Murray | PNG | 162 | 163 | 293 | 309 | 131 | 133 | 177 | 178 | 146 | 148 |
| 07MG216 | Lake Murray | PNG | 163 | 163 | 301 | 301 | 131 | 133 | 173 | 174 | 146 | 148 |
| 07MG217 | Lake Murray | PNG | 163 | 163 | 285 | 301 | 129 | 131 | 178 | 178 | 142 | 150 |
| 07MG218 | Lake Murray | PNG | 163 | 163 | 257 | 293 | 131 | 133 | 172 | 173 | 146 | 146 |
| 07MG219 | Lake Murray | PNG | 164 | 164 | 293 | 309 | 131 | 133 | 173 | 173 | 146 | 152 |
| 07MG220 | Lake Murray | PNG | 164 | 164 | 257 | 277 | 131 | 133 | 172 | 173 | 146 | 150 |
| 07MG221 | Lake Murray | PNG | 163 | 164 | 257 | 293 | 131 | 131 | 173 | 173 | 146 | 148 |

Table S2. Summary of Φ_ST_ values (below diagonal) and associated *p* values (above) in magpie goose *Anseranas semipalmata* (Latham 1798) by sites. Abbreviations: CYP – Cape York Peninsula; NWA –North-west Western Australia; PNG – Papua New Guinea.

|  | Big Red Lily Lagoon 2007 (CYP) | Billabong Sanctuary 2007 (FNQ) | Billabong Sanctuary 2008 (FNQ) | Hasties Swamp (FNQ) | Darwin (NT) | Kakadu (NT) | Kununurra (NWA) | Lake Murray (PNG) |
| --- | --- | --- | --- | --- | --- | --- | --- | --- |
| Big Red Lily Lagoon 2007 (CYP) | -- | 0.649 | 0.262 | 0.317 | 0.656 | 0.315 | 0.97 | 0.746 |
| Billabong Sanctuary 2007 (FNQ) | -0.006 | -- | 0.38 | 0.192 | 0.357 | 0.263 | 0.534 | 0.144 |
| Billabong Sanctuary 2008 (FNQ) | 0.005 | 0.002 | -- | 0.157 | 0.477 | 0.097 | 0.152 | 0.284 |
| Hasties Swamp (FNQ) | 0.002 | 0.007 | 0.006 | -- | -0.009 | 0.456 | 0.476 | 0.32 |
| Darwin (NT) | -0.005 | 0.003 | -0.001 | 0.977 | -- | 0.968 | 0.446 | 0.707 |
| Kakadu (NT) | 0.003 | 0.007 | 0.014 | 0 | -0.014 | -- | 0.302 | 0.203 |
| Kununurra (NWA) | -0.014 | -0.003 | 0.01 | -0.001 | 0 | 0.004 | -- | 0.668 |
| Lake Murray (PNG) | -0.006 | 0.012 | 0.004 | 0.002 | -0.005 | 0.007 | -0.005 | -- |
